# Frequent attenders of three outpatient health care schemes in Finland: characteristics and association with long-term sickness absences, 2016–2018

**DOI:** 10.1186/s12889-021-10866-x

**Published:** 2021-05-06

**Authors:** Riku Perhoniemi, Jenni Blomgren

**Affiliations:** The Social Insurance Institution of Finland, P.O. Box 450, 00056 KELA, Helsinki, Finland

## Abstract

**Objectives:**

Frequent attenders (FAs) impose a significant burden on service capacity and public health funding. Although the characteristics of the group and their risk for sickness absences (SA) have been studied, an understanding of FAs in different health care schemes is lacking. The aim of the study was to investigate FAs and their SA risk in the working-age population in public care, occupational health services (OHS) and private care schemes. The average number of SA days was also examined by diagnostic group.

**Setting and participants:**

Register data on the use of outpatient health care, sickness allowance spells and background characteristics (2015–2018) for 25–64 year old residents of the city of Oulu, Finland, (*n* = 91,737) were used. Subjects were categorized into non-attenders, non-frequent attenders and FAs (top decile of attenders) both for all outpatient health care and specifically for each care scheme in 2016. The number of sickness absence days was measured yearly in 2016, 2017 and 2018. The data were analyzed with descriptive methods and negative binomial regression models.

**Results:**

FAs consumed 31 to 44% of all visits depending on scheme in 2016. Frequent attendance was common among low socioeconomic groups in the public scheme, among lower non-manual employees and manual workers in OHS, and among entrepreneurs in the private scheme. FAs had a higher average number of SA days than others in each scheme, although group differences decreased from 2016 to 2017 and 2018. In public care, the adjusted effect of frequent attendance was strong especially for SA due to mental disorders (adjusted incidence rate ratio [IRR] for FAs 13.40), and in OHS for SA due to musculoskeletal disorders (adjusted IRR for FAs 8.68).

**Conclusion:**

In each outpatient health care scheme, frequent attenders pose a great challenge both by consuming services and through their increased risk of disability. FAs in different schemes have partially different characteristics and risks. Common patient registers covering various service schemes would enable an identification of FAs visiting multiple schemes and services. Better coordinated services are needed for public care FAs in particular.

## Introduction

Public health care funding and disability benefits are associated with enormous public expenditure in the OECD countries [1, 2]. Therefore, providing efficient health services and preventing disability are huge challenges for any nation. One key issue in meeting these challenges is to understand how health care attendance is associated with future disability.

The top 10% of health care attenders have been shown to consume 30 to 50% of total professional contacts [3–6]. Frequent use is often appropriate as severe sickness requires more treatment. However, frequent attenders (FAs) are a major challenge for public health funding and their needs pose a significant burden on service capacity.

Moreover, frequent health care attendance may often not be functional or as such meet clients’ needs. This is indicated by the high frequency of medically unexplained symptoms, health anxiety, negative life events and social problems among FAs [3, 5–10], and above all by the resilient nature of frequent attendance [3, 5, 6, 11, 12]. Foster et al. [13] and Vedsted and Olesen [14] have shown that frequent use of general practice health care is partly independent of the clients’ medical conditions. Indeed, frequent health care attendance tends to accumulate in distinct demographic and more vulnerable socioeconomic groups. FAs are more often women [6, 9, 11, 15, 16], older [5, 6, 11, 12, 15], without work [9, 14, 16], and have a lower occupational class [12, 16–18], and lower education [8, 14, 17–19]. Lower socioeconomic status may be associated with frequent attendance in primary care or general practice settings especially [8, 9, 16, 18, 19]. However, also among the employed, working in a manual field can increase the probability of frequent attendance of occupational health services (OHS) [3, 4, 17].

To date, there have been fewer studies on the consequences of frequent attendance than the characteristics of FAs. However, FAs may be at obvious risk for future occupational disability. Reho et al. [20] studied employees’ frequent attendance in a major Finnish provider of OHS. Adjusting for multiple covariates, FAs had more common and longer sickness absences compared to non-frequent attenders even 2 years after the frequent attendance episode. A study by Bergh et al. [7] showed similar results in Norwegian primary health care, as FAs had a three-fold probability of long-term sickness absence during a five-year follow-up compared to other attenders. In a study by Harkko et al. [17] on Helsinki city employees, a higher number of OHS visits predicted a higher risk for long sick leaves. As long sickness absence spells are a major predictor of permanent disability [21–24], understanding the association between frequent attendance and sickness absence is crucial.

Frequent attenders have mainly been studied in primary care and general practice settings, and to a lesser extent in OHS [12, 17, 20, 25]. There is a clear lack of studies comparing health care schemes. However, comparisons between schemes are pivotal in understanding the risks for, and consequences of, FA, as well as in creating new best practices. Different health care schemes – such as public, private, and OHS – are often utilized by different segments of the population [26–28]. Thus, also patterns and consequences of frequent attendance may vary between the users of each scheme.

In addition, diagnosis-specific knowledge of how health care attendance rate is associated with sickness absence is lacking. In Reho’s study [20], there was an association between the amount of OHS attendance and the probability of long-term sickness absence due to mental disorders. A higher frequency of mental disorders and mental problems has been found among FAs in primary care and general practice settings as well [5, 6, 8–10, 19, 29, 30]. Reho et al. [20] have called for comparative observations of diagnostic emphases from different health care schemes.

### Finnish health care schemes

There are three main outpatient health care schemes in Finland [31]. Public outpatient primary health care services, organized by municipal health centres, offer universal coverage for all residents. Though largely tax-funded, the use of these services is very affordable for the client at the point of delivery and does not depend on privately purchased medical insurance.

Second, occupational health services are the main provider of primary care services for the working population. All employees are entitled to at least employer-provided preventive care in OHS, but almost 90% of employers provided also primary care through OHS in 2013 [32]. Depending on the employer, the OHS can cover specialized care as well. The services are mostly provided by private companies. They are jointly financed by employers and by statutory social insurance fees, paid by the employers and employees together.

Third, use of private health care is state-supported via partial reimbursement, which covered around 17% of the costs in 2016 [33]. Because of strong public and OHS schemes, the role of the private scheme is rather small in Finland.

According to one estimate, the public scheme accounted for 42%, OHS for 33%, and the private scheme for 25% of total outpatient consultations with physicians in 2012 [34]. However, when looking at the annual proportion of users in each scheme, a study focusing on one Finnish city found that OHS were used by around 60%, public care by one third and private care by one fourth of city residents during 2013 [26].

Outpatient specialized care is offered both by the public and private schemes, and to a small extent by OHS. A little over one third of all Finns attended outpatient specialized care in 2018 [35].

The characteristics of health care attenders also vary greatly depending on the individual scheme. Higher socioeconomic status increases the probability of using OHS – either OHS exclusively or OHS complemented by private care. Respectively, the lower the socioeconomic status, the higher is the probability of not using any health care or using only public care [26].

### Research questions

Based on the results and limitations of previous studies, our research questions are:

1. What are the characteristics of frequent outpatient health care attenders in the public, OHS and private schemes?
2. How is frequent attendance associated with long-term sickness absence (overall and in the three health care schemes)?
3. Does the association between frequent attendance and sickness absence in the three health care schemes depend on the diagnostic group?

## Methods

### Study population

Register-based data for years 2015–2018 were collected from several registers for the total population of the city of Oulu, situated in Northern Finland [36]. Oulu is the fifth largest city of Finland, with a population of 198,525 inhabitants in 2016. On various demographic, socioeconomic or health care-related indicators, Oulu does not differ in any systematic way from Finland as a whole [36]. Individuals who were 25–64 years old, not retired at the end of year 2015, and residents of Oulu both at the end of 2015 and 2016 were included in this study (*n* = 91,737). Those receiving a pension at baseline were excluded, as pensioners are not entitled to sickness allowance.

### Data on outpatient health care schemes

Data on the use of outpatient health care were collected for the year 2016. Data on public health care attendance were obtained from the municipality of Oulu and from the Care Register for Health Care [37]. Visits to municipal health centres and outpatient visits to hospital specialized care were equally included in public care. In January 2016, 59% of the patients in northern Finland had the access to a physician’s appointment in public primary care (non-urgent treatment) in under 8 days (nationally 51% of the patients) [38]. Data on OHS attendance were gathered from the four largest OHS providers in Oulu (Terveystalo, Mehiläinen, Attendo and Työterveys Virta), estimated to cover around 92% of employees entitled to OHS [39]. Data on the use of private outpatient care were retrieved from the reimbursement registers of the Social Insurance Institution of Finland (Kela).

Active visits to all health care professionals, either face-to-face contacts, phone calls or virtual contacts, were included. Professionals included physicians, nurses, physiotherapists, psychologists and authorized nutritionists. Dental health care and laboratory visits were excluded to harmonize the data between the schemes. As public student health care visits were not fully available in the registers, we excluded residents under 25 years old from the study.

Since one visit was often registered as several different entries in the registers of the health care providers, it was not possible to reliably count the total number of visits by the same patient. Thus, as a proxy for the number of visits, we calculated the yearly number of attendance days for each subject in 2016. Attendance days were calculated for each scheme separately.

Based on the number of attendance days, a three-category variable was used to represent the attendance frequency. We used a common definition of FA as the top decile of all attenders [3–5, 7–10, 20, 30, 40]. Non-frequent attenders were defined as those who attended less than the FAs, but at least once in 2016. In addition, differing from several previous studies, we included non-attenders as well. This was done to widely detect differences in the scheme-specific amount of attendance and the association between attendance frequency and sickness absence. Thus, the following attendance frequency groups were used: non-attenders (non-As), non-frequent attenders (non-FAs), and frequent attenders (FAs). Groupings were done separately for visits in any scheme and for visits in each scheme.

Defining the attendance frequency groups separately for three schemes caused very little overlap between the groups. Virtually no one (*n* = 14) was FA in every scheme, while 0.4% (*n* = 348) were FAs both in the public scheme and in OHS, 0.2% (*n* = 158) were FAs both in OHS and in the private scheme, and 0.2% (*n* = 153) were FAs both in the public and private schemes.

### Measurement of sickness absence

Sickness absence (SA) was measured through compensated sickness allowance days. Kela can pay sickness allowance to non-retired persons aged 16–67 as compensation for loss of income due to inability to work because of sickness or impairment. The allowance can be paid after a waiting period of one (visit to a physician) plus nine working days, covered by the employer. Thus, the measure of sickness allowance captures only rather long-term SA spells.

A physician’s sickness certificate is a prerequisite for the allowance. Based on a certain diagnosis, the allowance can generally be granted for 1 year at most during 2 years’ time. After that, a disability pension may be considered.

Register data on sickness allowance spells from the years 2016–2018 were derived from Kela, including the start and end dates of compensated absences and the associated diagnoses. Primary diagnoses were categorized into three diagnostic groups according to the International Statistical Classification of Diseases, version 10 [41]: 1) Mental disorders, (F00-F99), 2) Musculoskeletal diseases (M00-M99), and 3) Other disorders. Mental disorders and musculoskeletal diseases are the main diagnostic groups of compensated SA in Finland [42]. The number of compensated SA days for the years 2016, 2017 and 2018 and in total were used as outcome measures.

### Covariates

Sex, age and marital status at the end of 2015 were examined as demographic characteristics and retrieved from Statistics Finland. Data on socioeconomic status and entitlement to reimbursement for medicine expenses, measured at the end of 2015, were retrieved from Kela registers. The sample was classified into four age groups (see Table 1). Marital status was classified into three groups. Socioeconomic status was measured as occupational class and education. Occupational class followed the classification of Statistics Finland [43]. The group “other” included the long-term unemployed and other persons outside the labour force. Education was categorized into upper tertiary, lower tertiary, secondary and basic level education. Entitlement to reimbursement for medicine expenses was used as a proxy measure for chronic disease [44]. These entitlements are part of the National Health Insurance system and guarantee the recipients access to medicines needed for the treatment of certain long-term diseases at a lower cost. Here, a division between no entitlement, entitlement to medicine(s) based on one disease, and entitlement to medicine(s) based on multiple diseases was used.

**Table 1:** The study population by covariates and outpatient health care use in 2016 (*n* = 91,737)

	n	%
Sex		
Male	46,773	51.0
Female	44,964	49.0
Age group		
25–34	27,797	30.3
35–44	24,876	27.1
45–54	21,711	23.7
55–64	17,353	18.9
Marital status		
Married	44,793	48.8
Unmarried	35,450	38.6
Divorced / separated / widowed	11,494	12.5
Educational level		
Upper tertiary	17,413	19.0
Lower tertiary	26,631	29.0
Secondary	38,565	42.0
Basic	9128	10.0
Occupational class		
Upper non-manual employee	21,672	23.6
Lower non-manual employee	26,103	28.5
Manual worker	15,737	17.2
Entrepreneur	5586	6.1
Other	22,639	24.7
Entitlement to reimbursement of medicine expenses		
No	74,078	80.8
Yes, 1 disease	13,836	15.1
Yes, multiple diseases	3823	4.2
Scheme use and overlap in 2016		
Only Public	19,701	21.5
Only OHS	17,564	19.1
Only Private	3572	3.9
Used all schemes	7038	7.7
Public + OHS	15,417	16.8
Public + private	6709	7.3
OHS+ private	5351	5.8
No use	16,385	17.9
**All**	**91,737**	**100.0**

### Statistical methods

The characteristics of the individual groups based on attendance frequency were examined through cross-tabulations. The association between attendance frequency and SA was examined through average SA days, but also with negative binomial regression models, adjusting for covariates. This method is suitable for count data with a right-skewed distribution [45]. Incidence rate ratios (IRRs) and predicted SA means with their 95% confidence intervals are presented. The analyses were conducted using Stata statistical software package 14.1.

### Ethical considerations

In Finland, an ethical review statement is not required for studies based solely on administrative register data [46]. We followed good scientific practice, data protection guidelines and ethical standards in collecting and analysing the data and in reporting the results. Permissions to use pseudonymised register data were obtained from the original data holders.

## Results

### Use of schemes

The overlap of attendance in the three examined health care schemes is presented in the lower part of Table 1. The most common attendance profiles were either to use only public care (22%), only OHS (19%) or to use both public care and OHS (17%). 18% had not used outpatient health care in 2016. In all, more than half (53%) of the study population had attended public health care in 2016, half (49%) had attended OHS, and one fourth (25%) had attended private health care.

Table 2 displays the attendance frequency groups by health care scheme. As the FAs were defined as the top decile of those who had attended care at least once, FAs constituted less than 10% of the whole sample. Thus, examining all outpatient health care use, 8% of the study population were defined as frequent attenders (with 21 or more visits in 2016, mean 38.3), while 74% were non-FAs and 18% non-attenders. *In public care*, 5% were frequent attenders (with 15 or more visits, mean 35.1), 48% non-FAs, and 47% non-attenders. *In OHS*, 5% were frequent attenders (with 17 or more visits, mean 24.3), 45% non-FAs, and 51% non-attenders. FAs *in private care* were a small group, 2% of the total study population, due to the infrequent use of the private scheme – 75% did not use the scheme in 2016. This also meant that FA status in private care was reached with as few as 5 visits (mean visits 10.8). 23% were non-FAs of private care.

**Table 2:** Attendance frequency groups and frequent attenders’ relative proportion of all outpatient health care visits by health care scheme in 2016

	All outpatient health care				Public				OHS				Private			
	non-A	non-FA	FA	Total	non-A	non-FA	FA	Total	non-A	non-FA	FA	Total	non-A	non-FA	FA	Total
n %	16,385 17.9	68,241 74.4	7111 7.8	91,737 100.0	42,872 46.7	44,326 48.3	4539 5.0	91,737 100.0	46,367 50.5	41,258 45.0	4112 4.5	91,737 100.0	69,067 75.3	20,996 22.9	1674 1.8	91,737 100.0
Attendance days a year	0	1–20	21–254		0	1–14	15–252		0	1–16	17–91		0	1–4	5–186	
Relative proportion of all outpatient health care by FAs (%)	0	64.1	35.9		0	56.5	43.5		0	69.5	30.5		0	63.5	36.5	

*non-A* non-attenders, *non-FA* non-frequent attenders, *FA* Frequent attenders

The proportions of all counted visits made by FAs in each scheme are also presented in Table 2. Examining all outpatient health care, they accounted for 36% of all counted visits. FAs of public care accounted for 44% of all public visits, FAs of OHS 31% of all OHS visits, and FAs of private care 37% of all private visits.

### Covariates and attendance frequency

Table 3 shows the attendance frequency groups according to covariates. Examining all outpatient health care, there was a clear association between covariates and attendance frequency. Frequent attendance was more common among females, older age groups, those divorced, separated or widowed, those with lower education, lower non-manual employees, those in the occupational class group “other”, and those with entitlement to reimbursable medication, especially based on multiple diseases. Non-attendance was more frequent among males, under 35-year-olds, unmarried individuals, those with basic education, entrepreneurs and those in the occupational class group “other”, and those without reimbursable medication.

**Table 3:** The probability of belonging to the attendance frequency groups by covariates and health care schemes

	All outpatient health care			Public			OHS			Private		
	non-A (%)	non-FA (%)	FA (%)	non-A (%)	non-FA (%)	FA (%)	non-A (%)	non-FA (%)	FA (%)	non-A (%)	non-FA (%)	FA (%)
Sex												
Male	24.6	70.4	4.9	56.3	40.3	3.4	53.9	43.2	2.9	83.8	15.0	1.2
Female	10.8	78.5	10.7	36.8	56.7	6.5	47.0	46.9	6.1	66.4	31.1	2.5
Age group												
25–34 y	22.3	72.2	5.5	45.2	49.6	5.3	60.3	37.8	1.9	80.6	17.9	1.5
35–44 y	18.5	74.5	7.0	49.3	46.2	4.4	48.9	47.3	3.8	76.3	21.7	2.0
45–54 y	16.4	75.0	8.6	50.0	45.4	4.6	45.1	48.9	6.0	72.7	25.7	1.6
55–64 y	11.8	77.0	11.3	41.4	53.0	5.7	44.1	48.3	7.6	68.6	29.1	2.4
Marital status												
Married	14.7	77.9	7.5	46.5	49.6	3.9	45.2	50.0	4.8	71.9	26.1	2.1
Unmarried	23.3	70.0	6.8	49.1	45.5	5.4	58.2	38.7	3.2	80.1	18.4	1.5
Divorced / separated / widowed	13.6	74.4	12.0	40.6	52.0	7.4	48.0	44.7	7.3	73.6	24.5	1.9
Educational level												
Upper tertiary	17.0	77.5	5.6	53.7	43.6	2.7	42.9	53.7	3.4	69.4	28.1	2.5
Lower tertiary	15.6	76.4	8.0	48.3	47.8	4.0	42.8	52.0	5.2	70.7	27.3	2.0
Secondary	18.5	73.1	8.4	43.6	50.5	5.9	55.0	40.2	4.8	79.2	19.4	1.5
Basic	23.3	68.3	8.5	42.3	49.7	8.0	68.7	27.9	3.4	83.6	15.1	1.4
Occupational class												
Upper non-manual employee	17.4	77.0	5.6	57.2	40.7	2.1	38.2	57.8	4.0	72.0	26.2	1.9
Lower non-manual employee	10.9	78.7	10.4	45.4	50.8	3.9	30.5	61.6	7.9	71.2	27.0	1.8
Manual worker	17.4	74.2	8.4	50.9	45.5	3.6	40.7	52.7	6.6	82.6	16.3	1.1
Entrepreneur	26.6	70.3	3.1	50.0	46.5	3.5	84.9	14.8	0.3	60.3	34.4	5.3
Other	24.5	68.1	7.4	34.7	55.2	10.2	83.8	15.5	0.7	81.8	16.8	1.5
Entitlement to reimbursement of medicine expenses												
No	20.7	73.8	5.5	51.6	45.0	3.4	52.1	44.5	3.5	76.4	22.0	1.6
Yes, 1 disease	6.7	78.7	14.6	29.0	61.1	9.9	44.2	48.1	7.7	70.3	27.2	2.6
Yes, multiple diseases	2.7	71.1	26.2	15.8	66.1	18.1	44.1	43.4	12.5	72.0	24.9	3.1
**All (%)**	**17.9**	**74.4**	**7.8**	**46.7**	**48.3**	**5.0**	**50.5**	**45.0**	**4.5**	**75.3**	**22.9**	**1.8**
**All (n)**	**16,385**	**68,241**	**7111**	**42,872**	**44,326**	**4539**	**46,367**	**41,258**	**4112**	**69,067**	**20,996**	**1674**

*non-A* non-attenders, *non-FA* non-frequent attenders, *FA* Frequent attenders

The higher proportion of FAs among females than males was seen in all schemes. The association between older age and frequent attendance was clearest in OHS, while the association between entitlement to reimbursable medication and frequent attendance was clearest in the public scheme. The association between socioeconomic covariates and attendance frequency depended strongly on scheme. In the public scheme, frequent attendance was much more common in the occupational class “other” and among those with basic or secondary education than in other groups. In OHS, by contrast, frequent attendance was common among lower non-manual employees and manual workers. In the private scheme, frequent attendance was common among entrepreneurs.

### Association between attendance frequency and long-term SA, 2016–2018

**Fig. 1:**
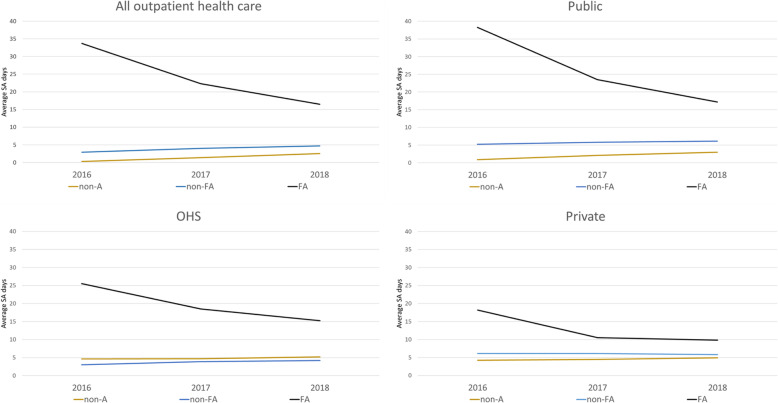
Average numbers of compensated SA days by health care scheme in 2016, 2017 and 2018. non-A = non-attenders, non-FA = non-frequent attenders, FA = frequent attenders

Figure 1 shows the average SA days in 2016, 2017 and 2018 by attendance frequency and health care scheme. For all outpatient health care, FAs had in 2016 on average significantly more SA days than non-FAs and non-attenders. The differences between the attendance frequency groups remained but diminished from 2016 to 2017 and 2018. The higher SA day average of FAs was seen in all schemes, as was the slightly diminishing temporal trend. Comparing the health care schemes, the difference of average SA days between FAs and other groups was largest in the public care scheme and smallest in private care. The difference between non-FAs and non-attenders was clearest, though small in the public care scheme, and only marginal in the two other schemes.

### Adjusted associations between attendance frequency and SA days by health care scheme and diagnostic group

The results of negative binomial regression models concerning the associations between outpatient health care attendance and the total number of SA days in 2016–2018 for different diagnostic groups are presented in Table 4. The first rows of each scheme-diagnosis-block show the unadjusted incidence rate ratios (IRR) for the expected number of SA days in 2016–2018 for non-FAs and FAs, with non-attenders as the reference group. The second rows show the IRRs adjusted for covariates. Adjusting for covariates somewhat lowered the IRRs. The third and fourth rows include the unadjusted and adjusted predicted means of SA days for each group, respectively.

**Table 4:** Sickness absence days 2016–2018 associated with attendance frequency in a negative binomial regression analysis model

	All outpatient health care			Public			OHS			Private		
	non-A	non-FA	FA	non-A	non-FA	FA	non-A	non-FA	FA	non-A	non-FA	FA
**All − cause SA**												
IRR (CI 95%. unadj.)	1.00	2.78 (2.57–3.01)	17.24 (15.17–19.61)	1.00	2.82 (2.65–3.00)	13.01 (11.89–14.98)	1.00	0.76 (0.72–0.81)	4.07 (3.50–4.74)	1.00	1.30 (1.21–1.40)	2.72 (2.16–3.43)
IRR (CI 95%. adj.)^a^	1.00	2.57 (2.38–2.79)	14.80 (13.01–16.84)	1.00	2.45 (2.30–2.60)	10.72 (9.30–12.35)	1.00	1.02 (0.96–1.10)	4.68 (4.02–5.44)	1.00	1.38 (1.28–1.48)	3.20 (2.55–4.01)
Predicted means (CI 95%. unadj.)	4.18	11.63	72.05	6.06	17.08	78.84	14.53	11.09	59.18	13.62	17.71	37.10
Predicted means (CI 95%. adj.) ^a^	3.84	9.89	56.87	5.74	14.05	61.57	10.47	10.73	49.00	10.9	15.01	34.84
**Mental SA**												
IRR (CI 95%. unadj.)	1.00	1.77 (1.50–2.09)	15.06 (11.47–19.78)	1.00	2.23 (1.96–2.54)	18.57 (13.78–25.02)	1.00	0.41 (0.36–0.46)	2.52 (1.83–3.46)	1.00	1.17 (1.00–1.37)	2.81 (1.72–4.59)
IRR (CI 95%. adj.) ^a^	1.00	2.10 (1.79–2.52)	17.18 (13.05–22.62)	1.00	1.96 (1.72–2.24)	13.40 (9.92–18.10)	1.00	0.64 (0.55–0.75)	4.07 (2.94–5.65)	1.00	1.31 (1.12–1.53)	3.53 (2.18–5.72)
Predicted means (CI 95%. unadj.)	1.69	2.99	25.48	1.83	4.08	33.92	5.62	2.28	14.16	4.2	4.92	11.80
Predicted means (CI 95%. adj.) ^a^	1.21	2.57	20.78	1.81	3.55	24.21	3.76	2.41	15.32	3.28	4.31	11.62
**Musculoskeletal SA**												
IRR (CI 95%. unadj.)	1.00	5.04 (4.33–5.87)	30.61 (23.94–39.15)	1.00	3.31 (2.94–3.73)	9.88 (7.52–12.99)	1.00	1.00 (0.89–1.13)	6.97 (5.22–9.29)	1.00	1.50 (1.31–1.73)	2.79 (1.79–4.35)
IRR (CI 95%. adj.) ^a^	1.00	4.12 (3.55–4.78)	24.13 (18.97–30.70)	1.00	2.92 (2.59–3.28)	8.59 (6.55–11.27)	1.00	1.46 (1.28–1.65)	8.68 (6.56–11.48)	1.00	1.50 (1.31–1.72)	3.50 (2.29–5.35)
Predicted means (CI 95%. unadj.)	0.73	3.66	22.20	1.79	5.72	17.67	3.61	3.61	25.13	3.98	6.00	11.10
Predicted means (CI 95%. adj.) ^a^	0.59	2.45	14.35	1.33	3.87	11.40	1.97	2.86	17.02	2.58	3.87	9.03
**Other diagnosis SA**												
IRR (CI 95%. unadj.)	1.00	2.83 (2.55–3.14)	13.84 (11.67–16.41)	1.00	2.89 (2.67–3.14)	11.14 (9.24–13.42)	1.00	0.98 (0.90–1.07)	3.75 (3.07–4.58)	1.00	1.25 (1.13–1.38)	2.61 (1.92–3.54)
IRR (CI 95%. adj.) ^a^	1.00	2.68 (2.42–2.98)	11.46 (9.65–13.61)	1.00	2.69 (2.47–2.92)	9.45 (7.83–11.41)	1.00	1.21 (1.10–1.32)	3.67 (3.01–4.48)	1.00	1.34 (1.22–1.48)	3.15 (2.33–4.24)
Predicted means (CI 95%. unadj.)	1.76	4.98	24.37	2.45	7.07	27.25	5.30	5.20	19.89	5.44	6.78	14.19
Predicted means (CI 95%. adj.) ^a^	1.62	4.34	18.51	2.23	6.00	21.12	4.05	4.88	14.87	4.38	5.87	13.77

*non-A* non-attenders, *non-FA* non-frequent attenders, *FA* frequent attenders

^a^ Adjusted for sex, age group, marital status, occupational class, educational level, and chronic disease

Compared to non-attenders and adjusted for covariates, FAs in all outpatient health care schemes combined had a 14.80-fold and non-FAs a 2.57-fold expected number of all-cause SA days in 2016–2018. In all outpatient health care, the effect of frequent attendance was strongest for SA due to musculoskeletal disorders, but strong for SA due to mental disorders as well. The same pattern can be seen in the adjusted predicted SA means as well.

The adjusted IRRs for the FAs were slightly stronger in public care, than in OHS or private care. In public care, the effect of frequent attendance was strong especially for SA due to mental disorders, and in OHS, especially for SA due to musculoskeletal disorders. In contrast to other schemes, in OHS the non-FAs had fewer SA days due to mental disorders than non-attenders. In the private scheme, the effect of frequent attendance on SA days was somewhat weaker than in other schemes but consistent regardless of the diagnosis.

## Discussion

In this study, we examined, first, the characteristics of frequent attenders (FAs) in three Finnish outpatient health care schemes and, second, the association of frequent attendance with sickness absence in the working-age population of one Finnish city. We assessed frequent attendance in the public, OHS and private care schemes. The FAs, here the top decile of attenders, accounted for over one third of all included attendance days. The disproportional use was clearest in public care, but the large cumulative consumption of the service capacity by a small number of clients was found in every scheme.

### Characteristics associated with frequent attendance

As in previous studies [6, 8, 9, 11, 12, 15–19], frequent attendance was most characteristic for females, older age groups, and those with a lower socioeconomic status. However, especially the socioeconomic characteristics of FAs depended on health care scheme, reflecting the different roles of the schemes in the Finnish health care system. In the public scheme especially, frequent attendance was common among those outside work or with only basic education. In general, lower socioeconomic groups in Finland are known to use mainly public services [26, 47]. Frequent attendance in the public scheme was also common for individuals with a chronic disease, and especially among those with multiple diseases. This may partly result from the tendency to refer long-term conditions to public care. The capacity of the public care scheme is thus heavily consumed especially by more financially vulnerable individuals and those with multi-morbidity. Although not studied here, social problems and negative life events are prevalent in these groups [5–7, 10]. These individuals are also often outside the scope of OHS services in Finland and in many cases cannot afford private care.

In the OHS scheme, frequent attendance was most common among lower non-manual and manual employees. Among employees, those with a lower socioeconomic position are most likely to use OHS, especially when adjusting for age and chronic disease [17, 26]. This generally higher attendance rate may be reflected in the higher proportion of FAs as well. Musculoskeletal disorders are both clearly more frequent [48] and pose a greater risk for disability retirement [49] among lower non-manual and manual employees than among upper non-manual employees. These disorders probably account for the higher FA rate in the OHS scheme as well. In addition, for some lower non-manual and manual employees frequent attendance may be caused by the need for a physician’s certificate from the first absence day. This kind of policy is more common for lower occupational classes in Finland [50]. Older age was associated with frequent attendance especially in OHS. In addition to the increasing incidence of health problems with higher age, this probably reflects the comprehensive services commonly available through OHS. OHS are available to most working-age Finns free of charge at the point of delivery, and employees with health problems have quite generous access to them according to medical need.

In private care, frequent attendance was particularly common among entrepreneurs. Obtaining OHS coverage is voluntary for entrepreneurs, and only 43% were estimated to do so in 2016 [51]. Thus, many entrepreneurs in great need of care may complement public care with private care.

### Attendance frequency and sickness absence days

Our main contribution is to shed light on the association between frequent health care attendance and subsequent sickness absence (SA) in the three health care schemes. FAs of each scheme had on average significantly more SA days than others in 2016 and this difference, albeit somewhat diminished, remained for the two follow-up years. The fact that the association between frequent health care attendance and SA days was strongest in 2016 can be explained by several reasons: One, because health care attendance was also measured for that year; two, because a physician’s sickness certificate is a prerequisite for sickness allowance; and three, because the dynamics between attendance and SA can work both ways. In the OHS scheme, the diminishing association over follow-up years may also be associated with adjusting the workload to better fit the individual capability of some employees. On the other hand, the association diminished similarly in all schemes. In all, frequent outpatient health care attendance, regardless of scheme, was a predictor of sickness absence for several prospective years.

Frequent attendance was associated with SA in all schemes but the strongest association was seen with public care. Combined with the lower socioeconomic status of public care FAs, the results indicate that especially the public care FAs have recurrent consultations without finding a solution to their primary problems, leading to a sick-leave later. This vulnerable group can include those with cumulative medically unexplained symptoms, health anxiety, negative life events and social problems [5–10]. Adjusted for covariates, public care FAs had on average 62 sickness allowance days in 2016–2018, reflecting the unmet needs and severity of health issues in this group. Interestingly, adjusting for covariates lowered the effect only little. This indicates that while an individual’s background affects the probability for frequent health care attendance, it does not appreciably shield individuals against the negative *outcomes* of excess attendance.

Further, our study shows that the association between frequent attendance and SA has different diagnostic emphases in different schemes. This demonstrates that studying the association in one scheme can lead to biased conclusions. Even though the risk for SA due to all three diagnostic groups was prominent for FAs in the public scheme, frequent attendance increased the expected number of SA days especially due to mental disorders. Individuals with low socioeconomic status are known to suffer from mental disorders more often than other socioeconomic groups [52]. As they are also more often public scheme FAs, it is understandable that their frequent attendance can later be accompanied by sickness absence due to mental disorders. The fact that adjusting for covariates reduced the effect that frequent attendance in the public scheme had for mental disorders especially suggests that this mechanism is true. In addition, a combination of comorbidity, social problems and negative life events may weaken public scheme FAs’ resources for coping with the initial mental health issues, possibly leading to future SA days. In our study the entitlement to reimbursed medicines based on multiple diseases - a marker for comorbidity- did indeed increase the probability for being a FA in the public scheme.

In OHS the association between frequent attendance and SA was strong especially for SA days due to musculoskeletal disorders. This is in line with Reho’s studies in which alongside mental disorders, especially musculoskeletal disorders as a consultation or a sickness allowance diagnosis have been associated with FAs in OHS [3, 4, 20]. Partly, this scheme-specific effect may reflect the use of the scheme in general. Musculoskeletal diseases and mental disorders are the main work-related reasons for OHS visits in Finland [53]. In private care, the association between frequent attendance and SA was not as strong as in the other two schemes, not even in the unadjusted models. This indicates that health issues are in general less severe for FAs of the private scheme than FAs of other schemes. Medical conditions might also be treated more quickly in self-paid private care, as the patient does not have to queue and doesn’t need a referral from primary care to specialized care provided in private care.

### Strengths and limitations

Our data on outpatient health care are exceptionally extensive, including all schemes relevant to the Finnish working-age population. To our knowledge, no studies on frequent attendance and its associations with SA with equivalent registers have been published. Our study included all residents aged 25–64 of the city of Oulu, and was based fully on register data deemed to be highly reliable and objective, with very little missing information, no self-report bias and no loss to follow-up. Furthermore, we were able to reliably calculate both the number of attendance days in each health care scheme and the precise length of sickness absence spells.

However, there are also some limitations. The findings do not cover the whole working-age population and are restricted to outpatient care only. We could not specify the proportions of primary and secondary care or the proportions of visits to the various professionals. This warrants caution in comparing the schemes. Due to the observational nature of the study, no causal effects can be shown between health care attendance and SA days. Naturally, the association does not run one way, but can work in both directions. For instance, an initial period of SA may be followed by rehabilitation or specialist care. However, it is probable that using health care affects the receipt of SA benefits. Health issues are mainly dealt with in health care, and only secondarily do they lead to long-term sickness absence. Also, a physician’s certificate is required to begin a period of sickness allowance. In future studies, both health care attendance and sickness absence should be followed over several years. Reho et al. [20] and Smits et al. [5] have compared occasional (1 year) and persistent frequent attendance, but a true longitudinal research setting is still lacking.

Finally, it has to be noted that the definition of FA as the top decile of individuals who attended outpatient health care can have implications for the results concerning attendance frequency and sickness absence days. Although FAs were defined similarly across all schemes in relative terms, the number of visits required to be classified as FA varied by scheme. As a sensitivity analysis we alternatively defined FAs in each scheme as individuals who attended health care on 10 days or more. The analysis did not change our key results: The effects (IRRs) of frequent attendance on SA were still strongest in the public scheme. Moreover, frequent attendance increased the SA days in the public scheme especially due to mental disorders, and in OHS especially due to musculoskeletal disorders. However, the effects for FAs strengthened in the private scheme in general, and frequent attendance now increased the SA days especially due to mental disorders. It is noteworthy however that the alternative definition of FAs used in the sensitivity analysis is problematic. As a result of standardizing the cut-off point of (ten) absolute attendance days, the relative proportions of FAs obviously diverged, respectively: in the private scheme, FAs comprised only 0.4%, but in all outpatient health care 25% of the total study population. We conclude that the decile-based relative method for defining FAs in different schemes is well-founded, and adequate in its ability to discriminate between groups [30].

### Practical implications

FAs are a vulnerable group [5–10] for whom poor health but also negative life events can be strong predictors of long-term sickness absence [7]. As the group may attempt to address unmet clinical and social needs with excess use of health care, they should be targeted with a wider service palette, including the possibility to access social services.

Many national systems have aimed for models of healthcare that can better coordinate and integrate various services, aiming to reduce fragmentation and add continuity of care (e.g. [54]). However, targeted interventions to influence morbidity, quality of life, and healthcare utilization among FAs have been modest in their effectiveness [55]. One method proposed is a personal case manager to ensure, coordinate, and integrate services for the patient [56]. A similar personal coordinator has been proved efficient in reducing public health centre visits among multimorbid clients [57]. A further key target for FAs could be to use frequent health care attendance as a marker for an assessment of need for rehabilitation. The fact that frequent attendance is a predictor for disability pension [58] supports such a targeted step.

Our study shows that the problem domain related to frequent attenders can be found in each health care scheme, and that there are FAs consuming capacity from multiple schemes as well. The strongest association with future SA days was found in this group. They cannot be identified without systematic patient registers shared by various schemes. The shared data base should include all health care, as well as social care visits regardless of scheme. In Finland, a recently developed electronic database, called Kanta services, makes sharing patient information (with a consent from a client) between health care providers possible. This may contribute to the continuity of care as well. In OHS, the continuity of care might be improved further by systematically guiding FAs to familiar OHS professionals.

More generally, the fact that lower socioeconomic groups are overrepresented among FAs showcases the strong link between socioeconomic status and health (e.g. [59, 60]). However it also indicates that polarization of health care attendance is not immune to change. Actively providing preventive care to the population with lower socioeconomic status may decrease cumulative somatic, mental and social problems, and therefore decrease the risk for excessive health care use later. As for employees, work environment factors such as occupational risks, physical and mental workload, and possibilities to influence one’s work affect the amount of OHS health care attendance [25] alongside the characteristics studied here. Our study showed that older employees, lower non-manual employees and manual workers are most often FAs in OHS, implying the potential of workplace adjustments for these employee groups.

## Conclusions

Frequent attenders pose a significant burden on service capacity in each outpatient health care scheme. The fact that frequent attendance is reflected in future sickness absence regardless of scheme further highlights the societal challenges related to frequent health care attendance. Given that not only public health care, but also sickness allowance is publicly financed [2, 61], the societal cost of substantial health care attendance is grave. FAs have a high average number of SA days in each outpatient health care scheme, but in public care especially due to mental disorders and in OHS due to musculoskeletal disorders. Excessive attendance is associated with low socioeconomic status in the public scheme, and there is a particular need for better coordinated services in this vulnerable group. In OHS, excessive attendance is associated with lower non-manual employee and manual worker status. These employee groups should be targeted with workplace adjustments to prevent frequent attendance and disability. In addition, patient registers including various service schemes would enable the identification of FAs who visit multiple schemes and services.

## Data Availability

Data cannot be shared publicly because strict restrictions apply to the availability of confidential individual-level register data. These analyses were conducted with permissions from third-party data holders for the current study. Permissions to obtain register data from the City of Oulu, from the Social Insurance Institution of Finland (Kela) and from the occupational health care providers may be applied for scientific research purposes from the Finnish Health and Social Data Permit Authority Findata (https://www.findata.fi/en/). A license to obtain register data from Statistics Finland may be applied for separately (https://www.tilastokeskus.fi/meta/tietosuoja/kayttolupa_en.html).

## Abbreviations

FAs: Frequent attenders
non-FAs: Non-frequent attenders
non-As: Non-attenders
SA: Sickness absences
OHS: Occupational health services
